# Self-Assembly of Amphiphilic Block Copolypeptoids – Micelles, Worms and Polymersomes

**DOI:** 10.1038/srep33491

**Published:** 2016-09-26

**Authors:** Corinna Fetsch, Jens Gaitzsch, Lea Messager, Giuseppe Battaglia, Robert Luxenhofer

**Affiliations:** 1 Functional Polymer Materials, Chair for Chemical Technology of Materials Synthesis, University Würzburg, Röntgenring 11, 97070 Würzburg, Germany; 2 Department of Chemistry, University College London, 20 Gordon Street, London WC1H 0AJ, United Kingdom; 3 Department of Chemistry, University of Basel, Klingelbergstrasse 80, 4056 Basel, Basel-Stadt, Switzerland

## Abstract

Polypeptoids are an old but recently rediscovered polymer class with interesting synthetic, physico-chemical and biological characteristics. Here, we introduce new aromatic monomers, *N*-benzyl glycine *N*-carboxyanhydride and *N*-phenethyl glycine *N*-carboxyanhydride and their block copolymers with the hydrophilic polysarcosine. We compare their self-assembly in water and aqueous buffer with the self-assembly of amphiphilic block copolypeptoids with aliphatic side chains. The aggregates in water were investigated by dynamic light scattering and electron microscopy. We found a variety of morphologies, which were influenced by the polymer structure as well as by the preparation method. Overall, we found polymersomes, worm-like micelles and oligo-lamellar morphologies as well as some less defined aggregates of interconnected worms and vesicles. Such, this contribution may serve as a starting point for a more detailed investigation of the self-assembly behavior of the rich class of polypeptoids and for a better understanding between the differences in the aggregation behavior of non-uniform polypeptoids and uniform peptoids.

For many decades the self-assembly of amphiphilic block copolymers has attracted much attention. Depending on the polymer architecture, molar mass and chemical composition this phenomenon yields structures of various morphologies^1^,^2^,^3^. To control the self-assembly, control over polymer structure is a prerequisite. Thus, the development of polymer assemblies was closely connected to the development of methods allowing control over polymer architecture, such as living polymerization and reversible deactivation radical polymerization^4^.

Classical morphologies are spherical micelles, worm-like micelles (also termed filomicelles) or polymer vesicles (also polymersomes) and have been investigated among other uses, as drug/gene delivery systems^2^,^5^,^6^,^7^,^8^,^9^,^10^,^11^. The solubilization/encapsulation of drugs with these morphologies in aqueous media brings several benefits^12^,^13^. Compared to micelles, which are able to solubilize hydrophobic compounds in aqueous solutions, polymersomes enable the solubilization of hydrophobic (inside the bilayer) and hydrophilic (in their interior) compounds due to the bilayer structure. In contrast to small-molecule aggregates such as liposomes, which are formed by the self-assembly of phospholipids, polymersomes exhibit higher colloidal stability, almost null critical aggregation concentrations and flexibility due to their mechanical and physical properties^14^. Many different block copolymers for the preparation of polymersomes have been described. Some structures are relatively simple such as the commercially available poly[(ethylene oxide)-*b*-(propylene oxide)]^15^ or poly[(acrylic acid)-*b*-(styrene)]^16^,^17^, poly[acrylic acid)-*b*-(butadiene)]^18^, poly[(ethylene oxide)-*b*-(styrene)]^19^ and poly[(ethylene oxide)-*b*-(butyl acrylate)]^20^,^21^. The use of more complex structures leads to polymersomes with special properties, which may promote the release of the encapsulated compounds. Degradable hydrophobic polymers, such as poly(lactic acid) or poly(ε-caprolactone) enables the preparation of bio-erodible polymersomes^22^, which are very attractive as drug delivery carriers. Further opportunities are pH^23^,^24^,^25^,^26^, oxidative^27^ or reductive^28^ sensitive polymers. Here, the sensitivity leads to the disassembly of the polymersomes.

The synthesis of well-defined polymers or block copolymers forms the basis for the preparation of polymersomes or other defined aggregates. Recently it has been shown that polypeptoids are accessible through a nucleophilic living ring-opening polymerization, which yields products with a very narrow molar mass distribution and end-group fidelity^29^,^30^,^31^,^32^. Furthermore, we reported that amphiphilic block copolypeptoids are able to self-assemble in aqueous solution and solubilize hydrophobic model compounds, but this study did not investigate the morphology of the resulting self-assemblies^33^. Polypeptoid based worm-like micelles and hydrogels based on such micelles were recently reported by Zhang and co-workers^34^,^35^. In addition to the synthetic and physico-chemical properties, polypeptoids exhibit interesting properties in *in vivo* and *in vitro* studies. Poly(*N*-methyl glycine) (polysarcosine, PSR) has already been described as non-immunogenic^36^,^37^. In more recent cell viability studies different polypeptoids were well tolerated by HepG2^33^ and HEL229^38^ cells at concentrations up to 10 g/L or 5 g/L, respectively. Therefore, polypeptoids may be interesting materials for drug delivery vehicles. Moreover, Kimura and co-workers investigated the *in vivo* retention in blood of a labeled so-called peptosome. These peptosomes consisted of a hydrophilic PSR and hydrophobic poly(γ-methyl l-glutamate) block and showed long circulation in rat blood comparable to PEGylated liposomes^39^. Labeled PSR showed a high escape ability from the mononuclear phagocyte systems^40^. These properties make the polypeptoids particularly suitable for the preparation of polymersomes, especially for biomedical applications. Vesicles from oligomeric peptoids, both as blocks and alternating monomer structure^41^,^42^ encouraged us to study the self-assembly of our amphiphilic block copolypeptoids^33^ in more detail. To the best of our knowledge the preparation of polymersomes consisting of block copolypeptoids, except PSR, has not been described in the literature up to now. For this work, we also introduce two new *N*-substituted *N*-carboxyanhydrides (NCA), *N*-benzyl glycine NCA and *N*-phenethyl glycine NCA, and their corresponding polymerization.

Here, we report on the first polymersomes from amphiphilic block copolypeptoids. We investigated the effect of variation of the polymer structure and different methods to prepare polymersomes. The resulting self-assembled structures were analyzed with dynamic light scattering and transmission electron microscopy.

Our results demonstrate that the polypeptoids are able to self-assemble into different morphologies depending on the hydrophilic fraction, hydrophobic moieties and self-assembly methods. This work may serve as a basis for further development of polypeptoids based polymersomes and filomicelles.

## Experimental Section

### Materials and methods

All substances for the preparation of monomers and polymers were purchased from Aldrich or Acros and were used as received unless stated otherwise. Benzonitrile (BN) was dried by refluxing over P_2_O_5_, benzylamine over BaO and petroleum ether over CaH_2_ under dry argon atmosphere and subsequent distillation prior to use. Water levels were determined using the Titroline^®^ 7500 KF trace (SI Analytics, Mainz, Germany). In general, solvents were used at water levels <30 ppm. The monomers were handled preferably in a glovebox (LabMaster 130, MBraun, Garching, Germany).

#### NMR spectroscopy

NMR spectra were recorded on a Fourier 300 (^1^H 300.13 MHz, ^13^C 75.48 MHz; Bruker Biospin, Rheinstetten, Germany) at room temperature (295 K). The spectra were calibrated using the solvent signals (CHCl_3_ 7.26 ppm, DMSO-*d*_6_ 2.50 ppm, TFA-d 11.5 ppm).

#### Gel permeation chromatography

Gel permeation chromatography (GPC) was carried out on a GPC system with UV-Vis detector SPD-6AV (Shimadzu, Duisburg, Germany) in hexafluoroisopropanol (HFIP; containing 4% ammonium trifluoroacetate) at room temperature using a PFG column (100 Å, 1000 Å; PSS, Mainz, Germany). The flow rate was set to 1 mL/min. The endpoint of the measurements was determined with an internal standard (toluene). Alternatively, GPC measurements were performed on a Polymer Standard Service (PSS, Mainz, Germany) System (MDS, RI detector) running under Win GPC software and using a 50 mm PFG precolumn and three 300 mm PFG columns (Mixed Bed PSS PFG linear M, 7 μm PSS, Mainz, Germany) for measurements in HFIP (containing 5 mmol/L ammonium trifluoroacetate). Columns were kept at 40 °C and the flow rate was set to 1 mL/min. Prior each measurement, samples were filtered through 0.2 μm PTFE syringe filters (Roth, Karlsruhe, Germany). Calibration of both GPC-Systems was performed using poly(methyl methacrylate) standards (PSS, Mainz, Germany) with molar masses from 0.8 kg/mol to 1600 kg/mol.

#### Transmission electron microscopy

Transmission electron microscopy (TEM) imaging was performed on a JEOL 2100 microscope operating at an acceleration voltage of 200 kV and equipped with a CCD Camera Orius SC2001 from Gatan.

The samples were mounted onto freshly glow-discharged carbon-coated copper grids (Agar Scientific, Essex, UK) at a concentration ranging from 0.5 to 1 g /L. After that, the grids were blotted with filter paper and immersed for 10 s into a phosphotungstic acid (PTA) solution at 0.75 wt% for negative staining. Then, the grids were blotted again and dried under vacuum for 1 min.

For PTA solution, 37.5 mg of PTA was dissolved in boiling distilled water (5 mL). The pH was adjusted to 7.0 by adding a few drops of 5 M NaOH under continuous stirring. The PTA solution was then filtered through a 0.2 μm filter.

#### Dynamic light scattering

Dynamic light scattering (DLS) running under ALV-7004 Correlator Software for Windows (Version 30.5.2) was performed with an ALV CGS-3 Multi Detection Goniometry System (ALV, Langen, Germany), equipped with a He-Ne laser (22 mW, λ = 632.8 nm) and eight fiber optical detection units including eight simultaneously working APD avalanche diodes. The measurements were conducted at scattering angles from 30° to 150° in steps of 5°. The samples were kept at 25 °C or 37 °C in a cell with temperature stability of ± 0.1 °C. All solutions (1 g/L) were filtered separately before measuring light scattering using 5.0, 1.2 or 0.8 μm syringe filters.

The resulting autocorrelation functions were double exponentially or stretched exponentially fitted to obtain the corresponding decay rates Γ. The plot of the decay rates against q^2^ show a linear dependence and the slope of this line corresponds directly to the diffusion coefficient according to Γ = Dq^2^. The corresponding hydrodynamic radii were calculated subsequently from the Stokes-Einstein-equation.

The distribution of r_H_ at 90° was obtained by a regularized inverse Laplace transformation algorithm of the correlation function, which is incorporated in the ALV software.

Some DLS measurements were carried out at 25 °C using a ZETASIZER Nano series instrument (Malvern Instruments, Greater Malvern, UK). The data were collected by the NIBS (non-invasive back-scatter) method using a He-Ne laser (4 mW, λ = 632.8 nm) and a fixed angle of 173°.

#### Preparation of polymersomes

Self-assembly of copolypeptoids in aqueous solution was investigated using either film rehydration or solvent switch method.

First, for film rehydration, block copolypeptoids were dissolved in a solvent, which is suitable for both blocks (aliphatic side chains: chloroform, aromatic side chains: HFIP). Afterwards the solvent was evaporated and the resulting polymer film was dried under reduced pressure for appox. 3 h. Finally, MilliQ water or buffer (pH 7.4) was added to the polymer film. The solution was stirred at room temperature for at least 7 d to ensure that polymer film is fully hydrated and detached from the glass wall. Initial DLS measurements were carried out after 7 d.

Self-assembled block copolypeptoids were also prepared with the solvent switch method. Here, the block copolypeptoids were dissolved in HFIP to give a 0.5 wt% polymer solution. After stirring overnight, water was added dropwise into the polymer solution at the rate of 5 μL/min (P5 (Table 1): 1 μL/min) until a 50 wt% water content was reached. The turbid solution was quenched immediately by slowly adding into an excess of water under continuous stirring. The final HFIP content in the solution was approx. 5 wt%. Finally, the organic solvent was removed from the solution by dialysis against MilliQ water for 5 times with solvent changes occurring every 3 h or longer. Prior the TEM measurements polymer solutions were characterized by DLS using the described ZETASIZER Nano series instrument.

## Synthetic Procedures

### Monomer Synthesis

#### Sarcosine N-carboxyanhydride (Sar-NCA)

Sar-NCA was synthesized in tetrahydrofuran (THF) using triphosgene as described previously^29^.

#### N-Butyl glycine-, N-pentyl glycine- and N-benzyl glycine-NCA

These monomers were obtained by a three-step synthesis from primary amines and glyoxylic acid using modified literature procedures^43^,^44^ and was described previously^29^,^31^.

Exemplary, the preparation of *N*-benzyl glycine-NCA was performed as follows.

- *N*-Benzyl glycine hydrochloride. Glyoxylic acid mono hydrate (20.56 g, 0.22 mol, 1 eq.) and benzylamine (11.77 g, 0.11 mol, 0.5 eq.) were added to CH_2_Cl_2_ (400 mL) and stirred at room temperature for 24 h. The solvent was evaporated, and 1 M HCl aqueous solution (400 mL) was added. The reaction mixture was heated under reflux for 24 h. The solvent was evaporated to yield a dark brown solid. Two recrystallization steps in methanol/diethyl ether (1/3, v/v) afforded the title compound as white powder (6.24 g, 28%). mp: 211–214 °C (lit. 213–214 °C)^45^.
-   ^1^H NMR (300 MHz; DMSO-*d*_*6*_): δ = 3.80 (2 H, s, COOH-C*H*_*2*_-NH), 4.15 (2 H, s, C_6_H_5_-C*H*_*2*_-) 7.48 (5 H, br, C_6_H_5_-), 9.52 (2 H, br, NH∙HCl), 13.77 ppm (1 H, br, COOH).
-   ^13^C{^1^H} NMR (125 MHz; DMSO-*d*_*6*_): δ = 46.17 (COOH-*C*H_2_-NH), 49.70 (C_6_H_5_-*C*H_2_-), 128.58 (-*C*H-C-*C*H-), 129.00 (-*C*H-CH-*C*H-), 130.12 (-CH-*C*H-CH-), 131.52 (-CH-*C*-CH-), 167.77 ppm (COOH).
- *N*-Benzyloxycarbonyl-*N*-benzyl glycine. *N*-benzyl glycine hydrochloride (5.76 g, 28.58 mmol, 1 eq.) was suspended in toluene (70 mL) and cooled to 0 °C. Sodium hydroxide (3.57 g, 87.56 mmol, 3 eq.) was dissolved in 70 mL water and added to the cooled suspension. After slowly adding of benzyl chloroformate (5.44 g, 30.96 mmol, 1 eq.) the solution was stirred for about 5 h and allowed to phase separate subsequently. The aqueous layer was separated and returned to the reactor and the pH value was adjusted to 1–2 using conc. HCl. The mixture was then extracted three times with 60 mL of ethyl acetate. The organic phase was dried over MgSO_4_ and the solvent was removed under reduced pressure to obtain brownish oil (7.29 g, 85%).
-   ^1^H NMR (300 MHz; CDCl_3_): δ = 3.96 (2 H, d, COOH-C*H*_*2*_-N-), 4.60 (2 H, d, C_6_H_5_-C*H*_*2*_-N-), 5.22 (2 H, d, C_6_H_5_-C*H*_*2*_-O-), 7.30 ppm (10 H, br, C_6_*H*_*5*_-CH_2_-O-, C_6_*H*_*5*_-CH_2_-N-).
- *N*-Benzyl glycine-NCA. To 13.23 g (47.36 mmol, 1 eq.) *N*-benzyloxycarbonyl-*N*-benzyl glycine were added 9.58 g (93.88 mmol, 2 eq.) acetyl chloride and 7.50 g (95.51 mmol, 2 eq.) acetic anhydride under dry argon atmosphere. The mixture was heated under reflux for 6 h at 70 °C. The excess of acetyl chloride and anhydride was removed under reduced pressure, yielding a brownish solid as crude reaction product. The crude product was dissolved in 20 mL dry chloroform and precipitated in 20 mL petroleum ether. Two recrystallization step in chloroform/petroleum ether (1/2, v/v) afforded the title compound as white powder (5.42 g, 82%). mp: 112 °C (lit. 114–115 °C)^43^.

  ^1^H NMR (300 MHz; CDCl_3_): δ = 3.95 (2 H, s, CO-CH_2_-N-), 4.57 (2 H, s, C_6_H_5_-C*H*_*2*_-), 7.33 ppm (5 H, br, C_6_H_5_-).

  ^13^C{^1^H} NMR (125 MHz; DMSO-*d*_*6*_): δ = 47.64 (C_6_H_5_-*C*H_2_-), 48.24 (CO-*C*H_2_-N-), 128.32 (-*C*H-C-*C*H-), 128.87 (-*C*H-CH-*C*H-), 129.33 (-CH-*C*H-CH-), 152.16 (-N-CO-O-), 165.07 ppm (-CH_2_-*C*O-O-).

#### N-Phenethyl glycine-NCA

- *N*-Phenethyl glycine hydrochloride. In a 1 L round-bottom flask 10.21 g (0.11 mol, 1 eq.) glyoxylic acid mono hydrate were dissolved in 260 mL dest. water. After complete dissolution 6.53 g phenethylamine (53.52 mmol, 0.5 eq.) was added. The reaction mixture was stirred at room temperature for 21 h. Afterwards, 270 mL 1 M HCl aqueous solution was added. The reaction mixture was heated under reflux for 24 h. The solvent was removed under reduced pressure to yield a white solid. Recrystallization in methanol/diethyl ether (1/2.5, v/v) afforded the title compound as colorless crystals (3.03 g, 26%). mp: 185–190 °C (lit. 184 °C)^46^.
-   ^1^H NMR (300 MHz; DMSO-*d*_*6*_): δ = 2.89 (2 H, m, C_6_H_5_-CH_2_-C*H*_*2*_-), 3.15 (2 H, m, C_6_H_5_-C*H*_*2*_-CH_2_-), 3.88 (2 H, s, COOH-C*H*_*2*_-NH-), 7.28 (5 H, br, C_6_H_5_-), 9.46 (2 H, s, NH∙HCl), 13.72 ppm (1 H, br, COOH).
-   ^13^C{^1^H} NMR (125 MHz; DMSO-*d*_*6*_): δ = 31.21 (C_6_H_5_-*C*H_2_-), 46.69 (C_6_H_5_-CH_2_-*C*H_2_-), 47.57 (COOH-*C*H_2_-N-), 126.68 (-CH-*C*H-CH-), 128.50 (-*C*H-C-*C*H-), 128.58 (-*C*H-CH-*C*H-), 137.12 (-CH-*C*-CH-), 167.96 ppm (COOH).
- *N*-Benzyloxycarbonyl-*N*-phenethyl glycine. ^1^H NMR (300 MHz; CDCl_3_): δ = 2.83 (2 H, m, C_6_H_5_-CH_2_-C*H*_*2*_-), 3.53 (2 H, m, C_6_H_5_-C*H*_*2*_-CH_2_-), 3.90 (2 H, d, COOH-C*H*_*2*_-N-), 5.12 (2 H, d, C_6_H_5_-C*H*_*2*_-O-), 7.25 ppm (10 H, br, C_6_*H*_*5*_-CH_2_-O-, C_6_*H*_*5*_-(CH_2_)_2_-N-).
- *N*-Phenethyl glycine-NCA. ^1^H NMR (300 MHz; CDCl_3_): δ = 2.95 (2 H, t, ^3^J_H,H_ = 7.0 Hz, C_6_H_5_-C*H*_*2*_-), 3.68 (2 H, t, ^3^J_H,H_ = 7.0 Hz, C_6_H_5_-CH_2_-C*H*_*2*_-), 3.80 (2 H, s, -CO-CH_2_-N-), 7.29 ppm (5 H, br, C_6_H_5_-).

  ^13^C{^1^H} NMR (125 MHz; CDCl_3_): δ = 33.86 (C_6_H_5_-*C*H_2_-), 45.03 (C_6_H_5_-CH_2_-*C*H_2_-), 49.67 (-CO-*C*H_2_-N-), 127.26 (-CH-*C*H-CH-), 128.49 (-*C*H-C-*C*H-), 129.08 (-*C*H-CH-*C*H-), 137.22 (-CH-*C*-CH-), 151.95 (-O-CO-N-), 165.26 ppm (-CH_2_-*C*O-O-).

#### Preparation of block copolypeptoids. Poly[(Sar)_50_-b-(N-BuGly)_58_], **P1**

In a glovebox, 0.19 g (1.68 mmol, 50 eq.) sarcosine-NCA was weighed into reaction vessel and 3.32 mL dry benzonitrile was added. After complete dissolution 33.2 μL of 1 M benzylamine in benzonitrile was added. The reaction mixture was stirred at room temperature under nitrogen atmosphere inside of the glovebox for 24 h. For analytical investigations of the first block, 150 μL were removed from the reaction mixture. Then 0.29 g (1.83 mmol, 58 eq.) of *N*-butyl glycine-NCA was weighted out and dissolved in 3.80 mL benzonitrile. The solution was added to the reaction mixture of the first block. Additional 7 d the reaction mixture was stirred under nitrogen atmosphere inside of the glovebox. The reaction mixture was precipitated into diethyl ether and isolated block copolypeptoid was dried under reduced pressure. After a further precipitation step the title compound was dissolved in methanol. The solvent was removed under reduced pressure and the polymer film was rehydrated in Millipore water and subsequently freeze-dried.

GPC (HFIP): M_n_ = 20.0 kg/mol, Ð_M_ = M_w_/M_n_ = 1.11 (PSar_50_); M_n_ = 27.2 kg/mol, Ð_M_ = 1.17 (block copolypeptoid).

^1^H NMR (300 MHz; TFA-*d*): δ = 1.00 (98 H, br, C*H*_*3*_-CH_2_-), 1.43 (69 H, br, CH_3_-C*H*_*2*_-), 1.74 (58 H, br, CH_3_-CH_2_-C*H*_*2*_-), 3.35 (185 H, br, CH_3_-(CH_2_)_2_-C*H*_*2*_-N, CH_3_-N), 4.60 (139 H, br, CO-CH_2_-N, C_6_H_5_-C*H*_*2*_-N), 7.32 ppm (5 H, br, C_6_H_5_-).

#### Poly[(Sar)_50_-b-(N-BuGly)_58_], **P2**

A further synthesis of the block copolypeptoids was carried out with another approach. After the addition of the second monomer the polymerization was allowed to proceed under reduced pressure (100 mbar) in a closed vessel at 70 °C for 3 d.

GPC (HFIP): M_n_ = 14.5 kg/mol, Ð_M_ = 1.09 (PSar_50_); M_n_ = 21.8 kg/mol, Ð_M_ = 1.17 (block copolypeptoid).

^1^H NMR (300 MHz; TFA-*d*): δ = 0.91 (72 H, br, C*H*_*3*_-CH_2_-), 1.34 (50 H, br, CH_3_-C*H*_*2*_-), 1.66 (44 H, br, CH_3_-CH_2_-C*H*_*2*_-), 3.16 (64 H, br, CH_3_-N), 3.40 (44 H, br, CH_3_-(CH_2_)_2_-C*H*_*2*_-N), 4.50 (91 H, br, CO-CH_2_-N, C_6_H_5_-C*H*_*2*_-N), 7.21 ppm (5 H, br, C_6_H_5_-).

#### Poly[(Sar)_50_-b-(N-PenGly)_52_], **P3**

The block copolypeptoid was synthesized with butylamine as initiator in the same procedure as described for P1.

GPC (HFIP): M_n_ = 15.2 kg/mol, Ð_M_ = 1.09 (PSar_50_); M_n_ = 24.3 kg/mol, Ð_M_ = 1.18 (block copolypeptoid).

^1^H NMR (300 MHz, TFA-*d*): δ = 0.95 (60 H, br, C*H*_*3*_-CH_2_-), 1.40 (78 H, br, CH_3_-(C*H*_*2*_)_2_-), 1.78 (34 H, br, CH_3_-(CH_2_)_2_-C*H*_*2*_-), 3.38 (97 H, br, CH_3_-(CH_2_)_3_-C*H*_*2*_-, C*H*_*3*_-N), 4.61 (75 H, br, CO-C*H*_*2*_-N, C_6_H_5_-C*H*_*2*_-N), 7.32 ppm (5 H, br, C_6_H_5_-).

#### Poly[(Sar)_50_-b-(N-BnGly)_45_], **P4**

The block copolypeptoid was synthesized with isobutylamine as the initiator in the same procedure as described for P2.

GPC (HFIP): 27.3 kg/mol, Ð_M_ = 1.03 (PSar_50_); M_n_ = 34.3 kg/mol, Ð_M_ = 1.10 (block copolypeptoid).

^1^H NMR (300 MHz, TFA-*d*): δ = 0.95 (6 H, d, ^3^J_H,H_ = 6.3 Hz, (C*H*_*3*_)_2_-CH-), 3.24 (178 H, br, (CH_3_)_2_-CH-C*H*_*2*_-, CH_3_-N), 4.57 (214 H, br, C_6_H_5_-C*H*_*2*_-N, CO-CH_2_-N), 7.16 ppm (140 H, br, C_6_*H*_*5*_-CH_2_-).

#### Poly[(Sar)_50_-b-(N-PhetGly)_41_], **P5**

The block copolypeptoid was synthesized with isobutylamine as the initiator in the same procedure as described for P2.

GPC (HFIP): M_n_ = 23.0 kg/mol, Ð_M_ = 1.05 (PSar_50_); M_n_ = 38.9 kg/mol, Ð_M_ = 1.07 (block copolypeptoid).

^1^H NMR (300 MHz, TFA-*d*): isobutyl group is not visible; δ = 2.95 (br, C_6_H_5_-C*H*_*2*_-), 3.44 (br, CH_3_-N), 3.97 (br, C_6_H_5_-CH_2_-C*H*_*2*_-) 4.78 (CO-CH_2_-N), 7.42 ppm (br, C_6_H_5_-).

## Results and Discussion

Block copolypeptoids with varying hydrophilic fractions were synthesized by sequential nucleophilic living ring-opening polymerization of *N*-carboxyanhydrides^29^,^31^. The synthesized block copolypeptoids consisted of a hydrophilic PSR, which is similar to the commonly used polyethylene glycol in that it is non-ionic, excellently soluble in water and a variety of organic solvents as well as biocompatible^32^. The chosen hydrophobic blocks comprised *N*-butyl glycine (P1 and P2), *N*-pentyl glycine (P3), *N*-benzyl glycine (P4) and *N*-phenethyl glycine (P5) which are comparable to well-known hydrophobic parts in polymersomes: *n*-butyl methacrylate (alkyl side chains) and styrene (benzene side chains), respectively. The characterization of all synthesized polypeptoids including molar masses and dispersities is summarized in Table 1. The degrees of polymerization as determined by ^1^H-NMR are slightly below the targeted values. In contrast, molar masses as determined by GPC are markedly overestimated which is explained the calibration using PMMA. It was thus important to use values obtained by NMR to determine the actual block-length ratio present in the final block copolypeptoids. In this respect, a hydrophilic fraction of 10–40 mol% is required for the preparation of polymersomes from amphiphilic block copolymers. We obtained for all polymers a value within the targeted range around 35–40%.

Subsequent to the synthesis and characterization of the block copolypeptoids, the self-organization of block copolypeptoids in MilliQ water and buffer was investigated with dynamic light scattering and transmission electron microscopy. The different structures of the hydrophobic side chains, aliphatic or aromatic, as well as the different hydrophilic fractions should have an influence on the aggregation of the polymers in the aqueous solution.

**Figure 1:**
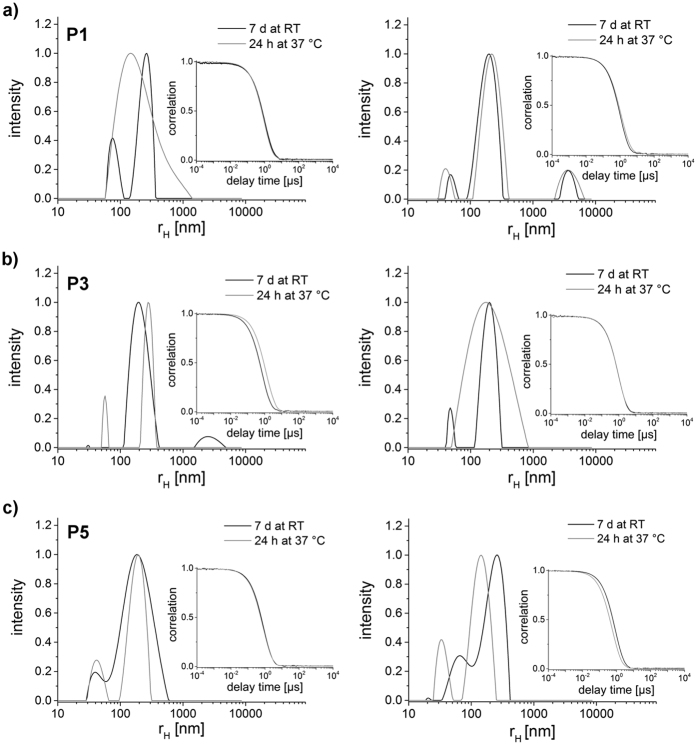
Dynamic light scattering measurements at 90° after different treatments in MilliQ water (left column) and buffer (right column) of (**a**) P1, (**b**) P3, and (**c**) P5.

The aqueous polymer solutions prepared by film rehydration were investigated at two points, 1) after stirring at RT for 7 d, (to ensure complete hydration) and 2) after further 24 h at 37 °C (to simulate physiological conditions). As expected, the resulting hydrodynamic radii of about 100 nm or larger are clearly above the size range of block copolypeptoid micelles^33^ (Table 2). We noted the resulting size distributions were often broad or hinted the presence of more than one size distribution in the sample (Table 2, Fig. 1). This is in line with previous studies, which have shown that the resulting particle sizes and size distributions of polymersomes are significantly influenced by the preparation method^10^. The method applied here, the film rehydration, often leads to broad and multimodal particle size distributions. Stirring the polymer solutions at 37 °C for 24 h did not change the hydrodynamic radii of the formed aggregates significantly but a general trend towards larger sizes was observed. At the same time, the heating led to an increased precipitation, especially for P4. This benzyl-containing polypeptoid was only poorly soluble at 37 °C in MilliQ water and buffer. Polypeptoids P1-P3 showed minor signs of precipitation only in buffer, which could be removed easily by filtration. Although this was not studied in more detail, these observations might point towards some thermoresponsive character of the block copolypeptoids investigated in the present study. Although the presence of vesicles is not proven by these values, it was clear that either polymersomes or other more complex non-spherical self-assembly structures were formed. The results were thus encouraging for further investigations.

**Figure 2:**
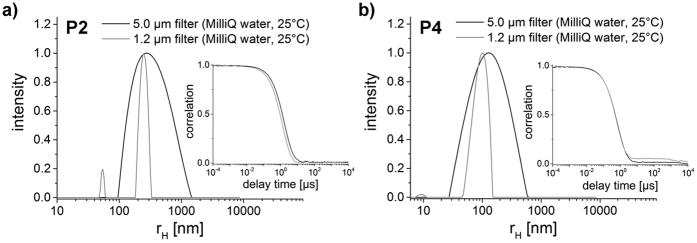
Particle size distributions and correlation functions of (**a**) P2 and (**b**) P4 in MilliQ water at 25 °C after the usage of filter with different pore sizes. Distributions result from the dynamic light scattering measurements at 90°.

As we found that temperature has only a minor influence, we investigated other parameters to influence the self-assembly structures formed. In contrast to the incubation at 37 °C the choice of the filter (pore size) had a significant influence on the resulting size distribution. Here, the polymer solution was filtered twice prior to the measurement (first with a larger filter (5 μm) and afterwards with a smaller size filter (1.2 μm)). It is important to note that we found no indications of material loss due to the filtration (visual inspection). The strongest change after repeated filtration could be observed for P2 and P4 (Fig. 2). The resulting size distributions were much more narrow, but the average hydrodynamic radii changed only slightly. This observation can be rationalized that during filtration, the aggregates are exposed to strong shear forces, which impacts in particular the bigger aggregates. This results in a narrower size distribution. The approach is similar to extrusion, which is a common method for the preparation of polymersomes and liposomes^10^,^47^,^48^. Here, the polymer solution passes through a non-porous polycarbonate filter several times. Mechanical forces act on the structures, narrowing the particle size distribution. Our results suggest that for the both polymers P2 and P4 the mechanical forces occurring during the filter process through a simple syringe filter are sufficient to obtain a similar effect. Comparable changes between the two different filters did not occur for the other three polymer solutions (P1, P3 and P5).

All results from DLS now pointed into the direction of self-assembly structures different from simple poly-mer micelles as the hydrodynamic radii (Table 2) were found clearly in the size range of vesicles. To get more conclusive results on the self-assembly behavior of our block copolypeptoids, the formed aggregates were also investigated by transmission electron microscopy (TEM). TEM allowed us to get visual confirmation of their morphology in a dried state. For this, we also investigated aggregates formed by the solvent switch method. In contrast to the film rehydration, the formation of aggregates is much faster with the solvent switch method, as the polymer is in solution to begin with. However, aggregates formed by this method may contain considerable amounts of organic solvent residues, which can be problematic for many applications, especially biomedical application. In case of film rehydration, the polymer has to go through the whole dissolution process from bulk solid to lyotropic liquid. This process depends mainly on the mutual diffusion of water into the bulk and vice versa by sub-diffusional processes^49^. For some selected block copolypeptoids (P2, P3 and P5) film rehydration and solvent switch as preparation methods were compared.

**Figure 3:**
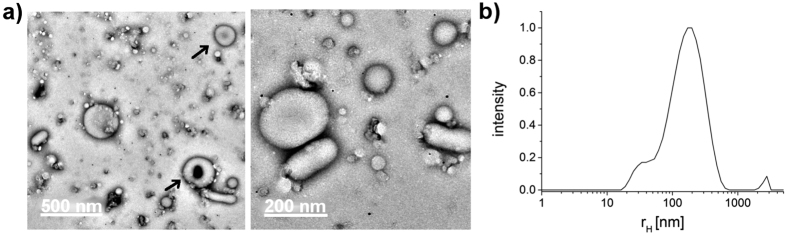
Aggregates of P1 in PBS (2 g/L) after film rehydration from chloroform. (**a**) TEM images of P1 vesicles and deformed vesicles are marked with arrows; (**b**) Particle size distribution of the corresponding polymer solution (P1). Distribution results from the dynamic light scattering measurement at 173° (zetasizer).

For the preparation of polymersomes from polymer P1 (butyl side chain) only the film rehydration was used. The polymer was dissolved in chloroform (c_P1_ = 4.75 g/L). After the removal of the solvent the resulting polymer film was rehydrated in phosphate buffered saline (PBS) (c_P1_ = 2 g/L). The rehydration resulted in micellar and vesicular morphologies after 6 weeks stirring (Fig. 3). The size of the vesicular structures ranges from 50 nm to 300 nm. This corresponds well with the size distribution obtained from the dynamic light scattering measurement of the polymer solution (Figs 1a() and 3(b)). The differences appear minor considering the different equipment employed. Among spherical vesicles also deformed vesicles, like slightly indented ones forming short tubes or donut-shaped “genus” vesicles, could be observed (Fig. 3(a)). This behavior is also known for methacrylic polymers^25^. Slight indentations of vesicles occur often due to pressure difference between the interior and the exterior of the vesicles during preparation^1^.

**Figure 4:**
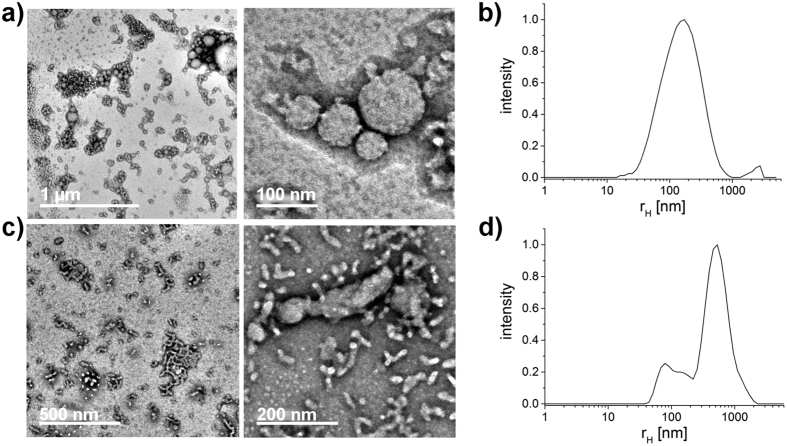
TEM images and particle size distribution of P2 polymer solutions after different preparation methods. Distribution results from the dynamic light scattering measurement at 173° (zetasizer). (**a**,**b**) Film rehydration from chloroform in PBS after 2 weeks stirring. (**c**,**d**) Solvent switch from HFIP to deionized water.

**Figure 5:**
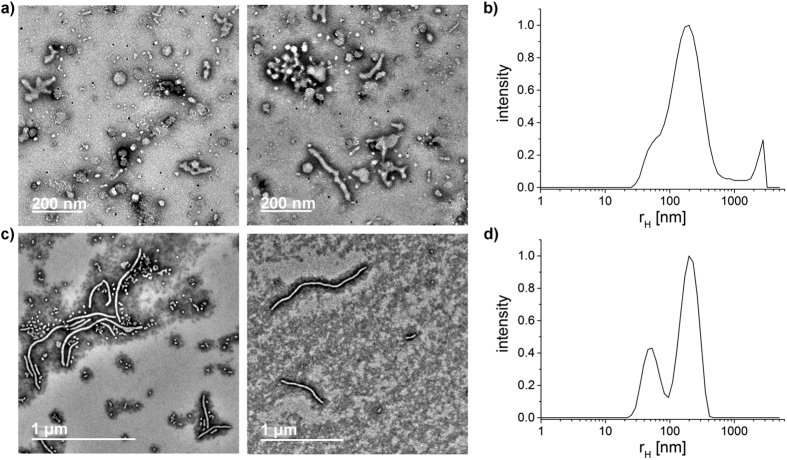
TEM images and particle size distribution of P3 polymer solutions after different preparation methods. Distribution results from the dynamic light scattering measurement at 173° (zetasizer). (**a**,**b**) Film rehydration from chloroform in PBS after 6 weeks stirring. (**c**,**d**) Solvent switch from HFIP to deionized water.

The aqueous solution of the similar block copolypeptoid P2 with a lower molar mass and slightly lower *f* was investigated after 2 weeks stirring. Here, the TEM images suggest interconnected vesicles with a slightly lower diameter compared to P1 (Fig. 4(a)). However, the particle size distribution based on DLS reveals a broad distribution in a similar size range like P1 (compare to Fig. 3(b). In case of P1 mainly free vesicles were measured during the DLS measurement, whereas the polymer solution of P2 also shows aggregates of several, yet individually smaller vesicles. This explains the similar particle size distributions for P1 and P2 measured by DLS. A longer stirring time in the case of P2 would probably lead to the separation of the vesicles and to a corresponding smaller particle size distribution. The smaller size may be attributed to the lower molecular weight of the block copolypeptoid. Self-assemblies of P2 were also prepared with the solvent switch from HFIP to deionized water. The resulting morphologies can be described as interconnected wormlike micelles. In addition, some vesicular structures could be discerned (Fig. 4c). Apart from the influence of the method, the different morphologies could be attributed to the solvent. In the rehydration method, the polymer was hydrated in PBS as opposed to deionized water. As the polypeptoids are essentially non-ionic polymers, we would not expect a major influence of the pH between PBS and deionized water. More likely, the salt concentration/ionic strength in the buffer increases the hydrophobic effects and promotes the aggregation via hydrophobic interactions^50^,^51^. Interestingly, Shen and Eisenberg have observed a change in morphology from spherical micelles to worm-like micelles to vesicles with decreasing solvent quality^52^. For the polypeptoids, the situation seems to be somewhat different, although are more detailed and systematic study will be necessary to clarify potential differences and why they occur. In the case of P3, this observation becomes more apparent. While in PBS a divers mixture of single and interconnected wormlike micelles, connected structures and vesicles are formed, the solvent switch to deionized water results in long (over 1 μm, d = 40 nm) and well-defined (in terms of worm-diameter) wormlike micelles after solvent switch (Fig. 5c) from HFIP to deionized water.

In addition to this, it is important to point out that the film hydration methods involve an evolution from solid phase block copolymers via hydration and consequent formation of lyotropic phases (i.e. hexagonal and cubic phase, lamellar phases, bicontinuous phase etc.) to the formation of isotropic phases i.e. vesicles or micelles^49^,^53^. This is quite a slow process where many metastable phases can be formed including multilamellar aggregates and tubular polymersomes^54^,^55^,^56^. On the other hand, the solvent switch process occurs via the nucleation and growth of unimer in solution and this process is critically dependent on the balance between the assembled state and unimers in solutions which at fast exchange rate can be depleted and hence structures do not mature into the final architecture but may be remaining in a kinetically trapped intermediate state^25^,^57^,^58^. It should be noted that Zhang and co-workers previously reported on worm-like micelles from block copolymers from linear and cyclic PSR-block-poly(*N*-decyl glycine)^34^,^35^. Worm-like micelles or filomicelles have also been discussed as highly promising drug delivery carriers. Discher and co-workers investigated the circulation of filomicelles and their spherical counterparts in rat blood^59^. Here, the circulation of filomicelles was reported to be about ten times longer. Together with the longer circulation of PSR in comparison to PEG, this makes filomicelles from amphiphilic block copolypeptoids interesting candidates in a drug delivery context. However, one must also note and caution that loading of self-assembled polymer based drug delivery systems may have a profound impact on their morphology^60^,^61^.

**Figure 6:**
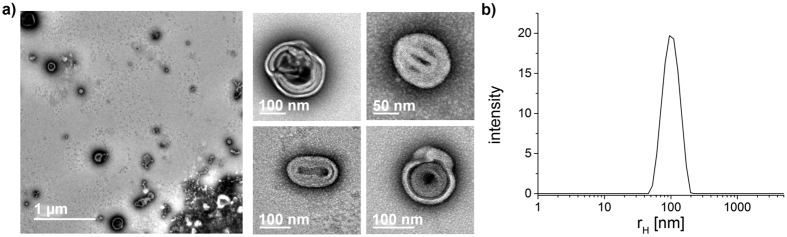
(**a**) TEM images of P5 vesicles; (**b**) Particle size distribution of the corresponding polymer solution (P5). Distribution results from the dynamic light scattering measurement at 173°.

Block copolymers with aromatic side chains in the hydrophobic part can be self-assembled into very stable aggregates due to the π-π interactions^62^. However, the hydrophobic block of these polymers is then also glassy, which leads to problems when attempting to rehydrate the polymer. Rehydration in aqueous solution can now result in the dispersion of bulk particles rather than in the formation of defined self-assembly structures. Indeed, this behavior could be observed for P4 and P5 for which the rehydration led to the formation of large and undefined aggregates (data not shown). The decrease of the polymer film thickness by decreasing the polymer concentration in the original solution from 5 g/L to 1 g/L did not improve the situation (attempted only for P5). Therefore, solvent switch from HFIP to deionized water was investigated for P5. From an initial 5 μL/min addition of water to the HFIP solution at which we also observed large and undefined aggregates, the water addition rate was reduced to 1 μL/min, which led to vesicular morphologies with a hydrodynamic radius around 100 nm (Fig. 6a). Although precipitation/aggregation could not be avoided completely (as also seen in the lower right corner of Fig. 6a), a considerable number of polymersomes were now obviously formed. Interestingly, although these vesicles are defined in size according to DLS, they consist of an oligolamellar shell. Such self-assemblies have also been termed onion-like vesicles. It appears that although the π-π interactions can stabilize the self-assembly structures formed, they also freeze them at an earlier stage, resulting the onion-like vesicles of collapsed lamellar structures. These vesicles may show some resemblance to vesicular structures comprising two oppositely charged amphiphilic and uniform copeptoids described by Zuckermann *et al*.^41^. The self-assembly in aqueous solution of sequence-specific peptoid polymers comprising alternations of charged peptoid units and *N*-phenethyl glycine units resulted in the formation of free-floating nanosheets after initial vesicle formation. Another recent example of similar oligolamellar vesicles was reported by Armes and co-workers for PEG-block-poly(2-hydroxypropyl methacrylate) samples^63^.

## Conclusion

We have investigated the preparation of polymersomes from amphiphilic block copolypeptoids with varying hydrophilic fraction *f* and hydrophobic building blocks. The block copolymers were synthesized via the nucleophilic living ring-opening polymerization of *N*-substituted *N*-carboxyanhydrides, including the novel aromatic monomers *N*-benzyl glycine NCA and *N*-phenetyl glycine NCA. The degrees of polymerization for the calculation of *f* were determined by ^1^H NMR. The resulting *f* were expected to be suitable for the formation of vesicular morphologies in aqueous solution. Dynamic light scattering measurements revealed for all investigated block copolypeptoids size distributions in the range of vesicular morphologies. An increase of the temperature up to 37 °C led to the precipitation of polymers, especially for the polymer with a benzyl side chain in the hydrophobic unit, hinting at a thermoresponsive character of these block copolypeptoids.

TEM images of the polymer solutions prepared with the rehydration method revealed the formation of vesicles among other morphologies for the polymers with aliphatic side chains in the hydrophobic unit. The polymers with glassy hydrophobic units led to very large aggregates. In case of the phenethyl side chain, a variation of the solvent switch method resulted in onion-like multilamellar vesicles.

For the first time, the formation of polypeptoids vesicles from amphiphilic block copolypeptoids is described. Here, hydrophobic units consisting of *N*-butyl glycine led to the best results, e.g. the most defined self-assembly morphologies although *f* is lower for the polymer with an *N*-pentyl glycine unit. Future and more detailed studies of polypeptoids with varying *f* and hydrophobic building blocks are necessary to elucidate the structure-property relationship of this class of biomaterials in more detail. Apart from the vesicular morphologies the polymer with *N*-pentyl glycine units formed very long and defined worm-like micelles, which could be interesting as a carrier for hydrophobic compounds such as drugs.

In summary, we report that amphiphilic block copolypeptoids can self-assemble into wide range of morphologies, including micelles, interconnected worms, filomicelles, vesicles and onion-like vesicles, depending on the polymer structure and the chosen route for self-assembly. Considering the rich side variability possible for polypeptoids and the relatively limited knowledge available for this class, the novel monomers and self-assembly structures open up new possibilities in this field.

## Additional Information

**How to cite this article**: Fetsch, C. *et al*. Self-Assembly of Amphiphilic Block Copolypeptoids – Micelles, Worms and Polymersomes. *Sci. Rep.* **6**, 33491; doi: 10.1038/srep33491 (2016).

## Figures and Tables

**Table 1:**
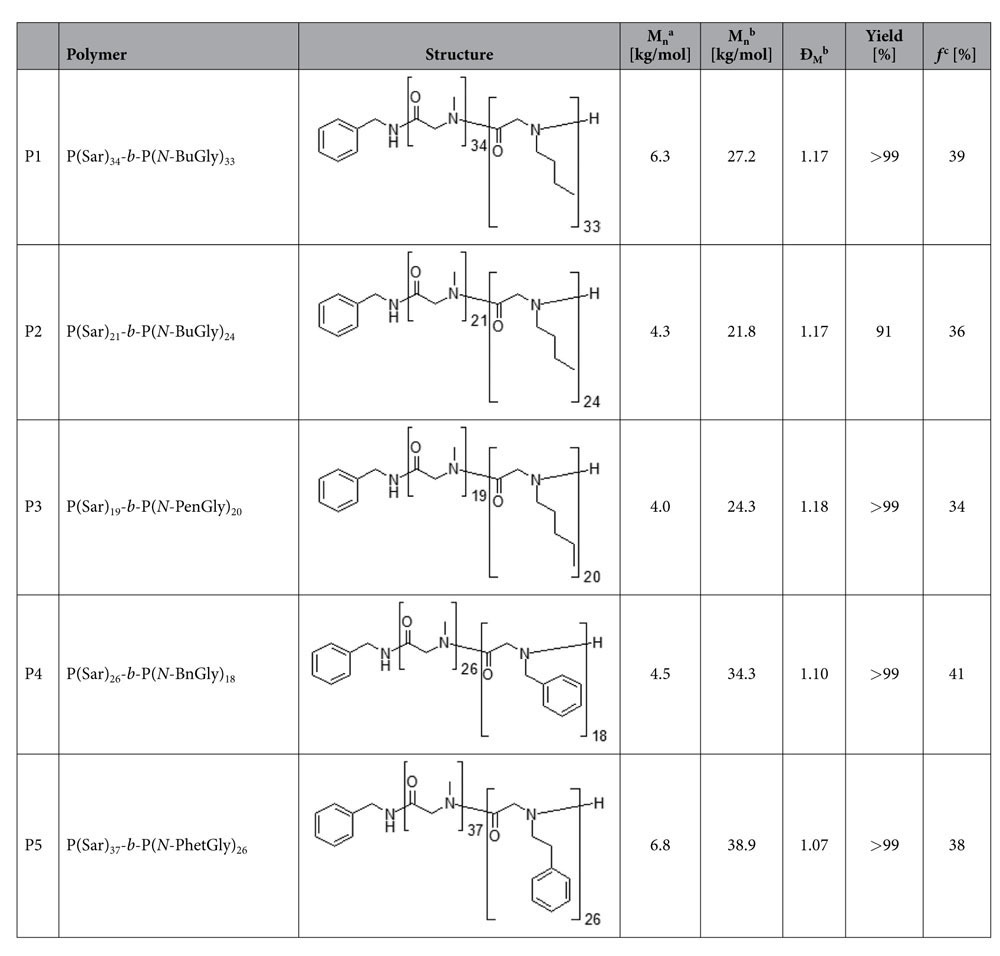
Analytical data of synthesized block copolypeptoids.

^a^As determined by end group analysis from ^1^H NMR spectroscopy. ^b^As determined by gel permeation chromatography. ^c^Hydrophilic fraction.

**Table 2:** Hydrodynamic radii of the block copolypeptoids in MilliQ water and buffer determine with dynamic light scattering.

Polymer	MilliQ H_2_O		Buffer (pH 7.4)	
	r_H_/nm (7 d)	r_H_/nm (37 °C for 24 h)	r_H_/nm (7 d)	r_H_/nm (37 °C for 24 h)
P1	206	169	118	108
P2	239	331	62	153
			215	
P3	115	91	62	152
		235	240	
P4	103	—	44	78
			183	343
P5	109	134	106	29
				138
