# Corrigendum: Self-Assembly of Amphiphilic Block Copolypeptoids – Micelles, Worms and Polymersomes

**DOI:** 10.1038/srep39441

**Published:** 2016-12-19

**Authors:** Corinna Fetsch, Jens Gaitzsch, Lea Messager, Giuseppe Battaglia, Robert Luxenhofer

Scientific Reports 6: Article number: 3349110.1038/srep33491; published online: 09 26 2016; updated: 12 19 2016

The Acknowledgements section in this Article is incomplete.

“This work was supported by the Fonds der Chemischen Industrie and the Free State of Bavaria. The authors gratefully acknowledge financial support by the German Plastics Center SKZ and Julius-Maximilians Universität Würzburg for start-up funding. Light-scattering experiments were possible through support of the Deutsche Forschungsgemeinschaft (INST 93/774-1 FUGG). RL, CF and JG thank the Deutsche Forschungsgemeinschaft (LU 1571/9-1 and GA 2051/1-1 respectively) for the financial support of their respective research stay at the University College London. GB and LM thank the support of the ERC-STG-MEVIC grant for funding the work and their salary.”

should read:

“This work was supported by the Fonds der Chemischen Industrie and the Free State of Bavaria. The authors gratefully acknowledge financial support by the German Plastics Center SKZ and Julius-Maximilians Universität Würzburg for start-up funding. Light-scattering experiments were possible through support of the Deutsche Forschungsgemeinschaft (INST 93/774-1 FUGG). RL, CF and JG thank the Deutsche Forschungsgemeinschaft (LU 1571/9-1 and GA 2051/1-1 respectively) for the financial support of their respective research stay at the University College London. GB and LM thank the support of the ERC-STG-MEVIC grant for funding the work and their salary. This publication was funded by the German Research Foundation (DFG) and the University of Wuerzburg in the funding programme Open Access Publishing.”

